# Somatic Representation of Emotional Problems among Native Kyrgyz Speakers

**DOI:** 10.1192/j.eurpsy.2024.1082

**Published:** 2024-08-27

**Authors:** E. Molchanova

**Affiliations:** Psychology, American University of Central Asia, Bishkek, Kyrgyzstan

## Abstract

**Introduction:**

The somatization problem has been one of the most acute in mental health for half a century (Kirmayer, L., 2000). Patients with somatic complaints turn to specialists in various fields but rarely to psychologists and psychiatrists, although the connection between bodily suffering and psychological difficulties sometimes lies on the surface (Molchanova E., 2016). In the last twenty years, the mechanisms of somatization have been considered by several disciplines, one of which is cultural psychiatry, which has become relevant.

Unfortunately, most of the research focuses on the cultural characteristics of migrants living in the United States (Groleau, D. and Kirmayer, L. 2004). There needs to be more research on the cultural features of somatization in Kyrgyz culture.

**Objectives:**

The goal of the study is to discover the distinctive features of the process of somatization in Kyrgyz culture

The objectives are:

To create a vocabulary of somatic phrases and idioms used to represent somatic problems to find the most commonly used somatic idioms for emotional complaints by native speakers of the Kyrgyz language.

To describe the mechanism of transformation of the emotional symptom into a specifically located and presented somatic complaint.

**Methods:**

The research used a mixed, qualitative, and quantitative design.

The first stage is qualitative, including ten semi-structured interviews with linguists, culturologists, historians, and specialists in folk art.

The second stage included four focus groups (12 people in each group) with a follow-up analysis. The recruitment of respondents was carried out through social networks, announcements, and the snowball method.

The third stage was quantitative. With the help of the dictionary compiled at the first stage, 250 participants ranked the frequency of somatic idioms, which were used to express the emotional problems

**Results:**

There have been found more than 200 somatic idioms, which are used to present emotional problems. The most frequent ones describe the heart, liver, and joints. Heart metaphors are associated with despair and anxiety, joints - with depression, and liver metaphors - with some personality characteristics, such as conformity and kindness. The created map of somatic representation of emotional problems shows the most frequent localization of somatic symptoms in disorders featuring somatic symptoms in native Kyrgyz speakers.

**Conclusions:**

There is a culturally shaped specifics of somatic representations of emotional problems among native Kyrgyz speakers. More research needs to be conducted to interpret the nature of people’s emotional problems represented by somatic symptoms.

**Disclosure of Interest:**

None Declared

